# Development of a Graphene Oxide-Incorporated Polydimethylsiloxane Membrane with Hexagonal Micropillars

**DOI:** 10.3390/ijms19092517

**Published:** 2018-08-25

**Authors:** Yi-Ying Lin, Yueh Chien, Jen-Hua Chuang, Chia-Ching Chang, Yi-Ping Yang, Ying-Hsiu Lai, Wen-Liang Lo, Ke-Hung Chien, Teh-Ia Huo, Chien-Ying Wang

**Affiliations:** 1 Institute of Pharmacology, School of Medicine, National Yang-Ming University, Taipei 11217, Taiwan; s19609005@gm.ym.edu.tw (Y.-Y.L.); g39005005@gmail.com (Y.C.); chuangjenhua5@gmail.com (J.-H.C.); tihuo@vghtpe.gov.tw (T.-I.H.); 2 Department of Medical Research, Taipei Veterans General Hospital, Taipei 11217, Taiwan; d49405004@gmail.com; 3 Department of Biological Science and Technology, National Chiao Tung University, Hsinchu 30010, Taiwan; ccchang01@faculty.nctu.edu.tw; 4 Center For Intelligent Drug Systems and Smart Bio-devices (IDS2B), National Chiao Tung University, Hsinchu 30010, Taiwan; 5 Institute of Physics, Academia Sinica, Taipei 11217, Taiwan; 6 Department of Neurosurgery, Tri-Service General Hospital, National Defense Medical Center, Taipei 11490, Taiwan; molly0103@gmail.com; 7 Institute of Oral Biology, National Yang-Ming University, Taipei 11217, Taiwan; wllo@vghtpe.gov.tw; 8 Division of Oral and Maxillofacial Surgery, Department of Stomatology, Taipei Veterans General Hospital, Taipei 11217, Taiwan; 9 Department of Dentistry, School of Dentistry, National Yang-Ming University, Taipei 11217, Taiwan; 10 Department of Ophthalmology, Tri-Service General Hospital, National Defense Medical Center, Taipei 11490, Taiwan; yred8530@gmail.com; 11 Department of Medicine, Taipei Veterans General Hospital, Taipei 11217, Taiwan; 12 Department of Critical Care Medicine, Taipei Veterans General Hospital, Taipei 11217, Taiwan; 13 Division of Trauma, Department of Emergency Medicine, Taipei Veterans General Hospital, Taipei 11217, Taiwan; 14 School of Medicine, National Yang-Ming University, Taipei 11217, Taiwan

**Keywords:** polydimethylsiloxane, graphene oxide, micropillar, retinal pigment epithelial cells

## Abstract

Several efforts have been made on the development of bioscaffolds including the polydimethylsiloxane (PDMS) elastomer for supporting cell growth into stable sheets. However, PDMS has several disadvantages, such as intrinsic surface hydrophobicity and mechanical strength. Herein, we generated a novel PDMS-based biomimetic membrane by sequential modifications of the PMDS elastomer with graphene oxide (GO) and addition of a hexagonal micropillar structure at the bottom of the biomembrane. GO was initially homogenously mixed with pure PDMS and then was further coated onto the upper surface of the resultant PDMS. The elastic modulus and hydrophilicity were significantly improved by such modifications. In addition, the development of hexagonal micropillars with smaller diameters largely improved the ion permeability and increased the motion resistance. We further cultured retinal pigment epithelial (RPE) cells on the surface of this modified PDMS biomembrane and assayed its biocompatibility. Remarkably, the GO incorporation and coating exhibited beneficial effect on the cell growth and the new formation of tight junctions in RPE cells. Taken together, this GO-modified PDMS scaffold with polyhexagonal micropillars may be utilized as an ideal cell sheet and adaptor for cell cultivation and can be used in vivo for the transplantation of cells such as RPE cells.

## 1. Introduction

Since the emergence of regenerative medicine and tissue transplantation, investigators have continuously focused on researching the restoration or regeneration of defective tissues [1]. Transplantation or engineering of tissues require three key components: cells, scaffolds, and cell signals [2]. The transplanted cells must attain the appropriate phenotypes in order to recover specific functions and physiology in the target organs of recipients. Cell signals, such as growth factors, cytokines, or bioactive molecules, are crucial for the maintenance or induction of cell growth and normal biological functions. The scaffolds house the cells, provide structural support for cell attachment, and substitute for the impaired tissue microenvironment. The physical interaction between transplanted cells and the interface of biomaterial is regarded as a key factor affecting the cellular phenotype, behavior, and consequent success of transplantation. To promote the interaction among cells, scaffolds, and cell signals, the suitable materials of biomimetic scaffold for microenvironment reconstruction have been extensively studied in different transplanted cells [3].

The scaffold constitutive materials in tissue engineering can be categorized into natural (e.g., collagen, gelatin, silk, cellulose, and chitin/chitosan) and synthetic materials (poly(ε-caprolactone), poly(ethylene glycol)(PEG), poly(lactic acid)(PLA), and poly(lactic-co-glycolic acid)(PLGA)). Polydimethylsiloxane (PDMS), a silicone-based and rubbery elastomer, is one of the polymeric materials widely used in the biomedical fields, including in biochip, biosensor, microfluidic channel, and mechanosensory biology applications [4,5,6,7]. The extraordinary characteristics of PDMS include low toxicity, chemical inertness, thermal stability, low autofluorescence, nanoscale precision, tunable mechanical strength/elasticity, optical transparency, biostability, oxygen solubility, facile fabrication, and inexpensiveness [7,8,9,10,11,12]. However, the intrinsic hydrophobicity of the PDMS surface substantially retards cell adhesion, resulting in distorted cell morphology, cell agglomeration, and cell detachment [5,13,14]. This limitation makes PDMS elastomers unsuitable for long-term cell cultivation. Besides, a laminin-modified PDMS biomimetic showed low mechanical strength in our previous study on transplantation of human induced pluripotent stem cell-derived retinal pigment epithelial cells (hiPSC-RPE cells) [4]. This low mechanical strength of the PDMS scaffold may hinder its applicability for transplantation, because such a scaffold is unable to self-stand and curl up. Therefore, surface modification that decreases the intrinsic hydrophobicity and/or enhances mechanical strength is required for the development of a long-term favorable interface. 

Several filler materials, such as metallic nanoparticles, polymeric fibers, carbon nanotubes (CNT), and ceramics, have been incorporated into PDMS scaffolds or other polymers to improve their mechanical properties [15,16,17]. Graphene, a monolayer of sp2-bonded carbon atoms hexagonally arranged into a two-dimensional honeycomb lattice [18], has attracted much attention over the past few years [19,20,21]. The excellent electrical conductivity and charge carrier mobility of graphene has enabled this material to be beneficial for the development of electronic devices [22], and now it is widely used in several biomedical applications, including biosensors, drug and gene delivery, cell imaging devices, electrical stimulation of cells, phototherapy for cancer, bioassays [23,24,25,26,27], and as the substrate for anchorage-dependent cells [24,28,29,30,31]. Graphene oxide (GO), a graphene nanosheet with oxygen-containing groups [32], has been reported to improve the properties of bioscaffolds for tissue transplantation, in particular for bone tissue [33,34,35,36,37,38,39]. In addition, GO-enriched bioscaffolds have several advantages, including low cytotoxicity [37] and higher physical strength [38], and exhibit promising biocompatibility [36]. Substrates coated with GO have also been demonstrated to provide excellent interfaces for cell–scaffold interaction in vitro [31]. In the present study, we sought to develop a novel PDMS-based bioscaffold with GO modifications, including the GO incorporation and coating onto the scaffold. Meanwhile, we added a substantial amount of hexagonal micropillars at the bottom of scaffold to enhance its motion resistance. These modifications conferred the properties of low intrinsic hydrophobicity, higher mechanical strength, and higher coefficient of friction of the bottom surface to the PDMS elastomer, thus enabling it to serve as a highly biocompatible scaffold for long-term cell cultivation and cell transplantation. 

## 2. Results

### 2.1. Manufacture of PDMS/GO Membrane

**Figure 1:**
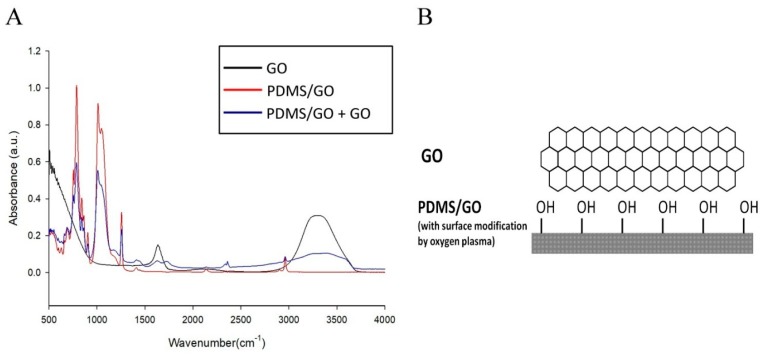
The FTIR analysis of graphene oxide (GO)-coated Polydimethylsiloxane (PDMS) incorporated with graphene oxide (PDMS/GO + GO) showed both PDMS- and GO-characteristic peaks. Peaks at 1100 cm^−1^ represent the Si–O group. (**A**) FTIR analysis of PDMS/GO, GO, and GO-coated PDMS/GO. The FTIR spectra profile demonstrated that GO contained hydroxyl group, C–C, and C=C stretching, while PDMS/GO is characterized by the Si–O–Si spectra. PDMS/GO contained hydroxyl, C–C, C=C, and Si–O–Si groups with different absorbance intensity. (**B**) The predicted chemical structure of PDMS/GO with GO coating (PDMS/GO + GO).

In this study, we attempted to develop a modified PDMS-based biomimetic scaffold with GO enrichment and the addition of hexagonal micropillars. By these modifications, we aimed to change several physical characteristics of PDMS, including hydrophobicity, mechanical strength, and friction force, and further improve its suitability for biomedical applications. After manufacturing of the PDMS/GO membrane by forceful agitation of PDMS and GO solution as described in the Materials and Methods, we analyzed the chemical composition of various samples, including PDMS/GO, GO alone, and GO-coated PDMS/GO, by using the Fourier transform infrared (FTIR) spectroscopy (Figure 1A). The GO-only sample produced two peaks: one strong peak at 3433 cm^−1^ due to O–H stretching and a small shoulder at 1577 cm^−1^ due to the strong C=C bond stretching vibration. The characteristic features of the PDMS spectra showed a band of asymmetric stretching of Si–O–Si at around 1100 cm^−1^.

Spectra peaks from the PDMS/GO sample showed the asymmetric stretching of Si–O–Si around 1100 cm^−1^, indicating the substantial excess of PDMS substrate. At the same time, we observed the aforementioned characteristic spectra of GO in the PDMS/GO sample with a slightly reduced absorbance intensity, indicating the successful incorporation of GO into the PDMS membrane. As for the GO-coated PDMS/GO (PDMS/GO + GO), an obvious absorption at 3433 cm^−1^ was observed, consistent with the observations of GO (Figure 1A). Therefore, the chemical composition and covalent bonding of the fabricated materials were predicted to be as in the structure shown in Figure 1B. The PDMS rubbery silicone elastomer is composed of the Si–C bonding, which constitutes the backbone of the PDMS elastomer, and GO-interconnected hexagons coated on its surface. 

### 2.2. Comparison of the Characteristics of Pure PDMS and Other GO-Modified PDMS Elastomers 

**Figure 2:**
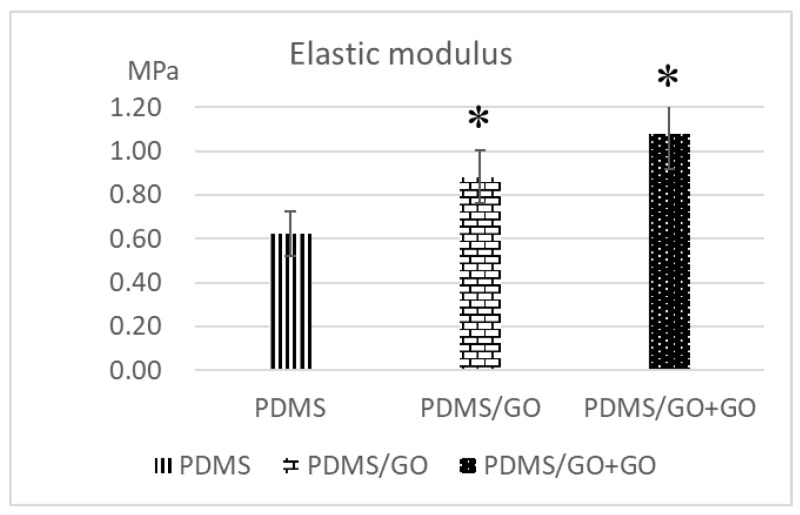
Measurement of elastic modulus of pure PDMS and GO-modified PDMS. The elastic moduli of pure PDMS, PDMS/GO, and PDMS/GO + GO were analyzed by the universal testing machine. The results are the mean ± SD of three independent experiments. * *p* < 0.05 vs. PDMS.

**Figure 3:**
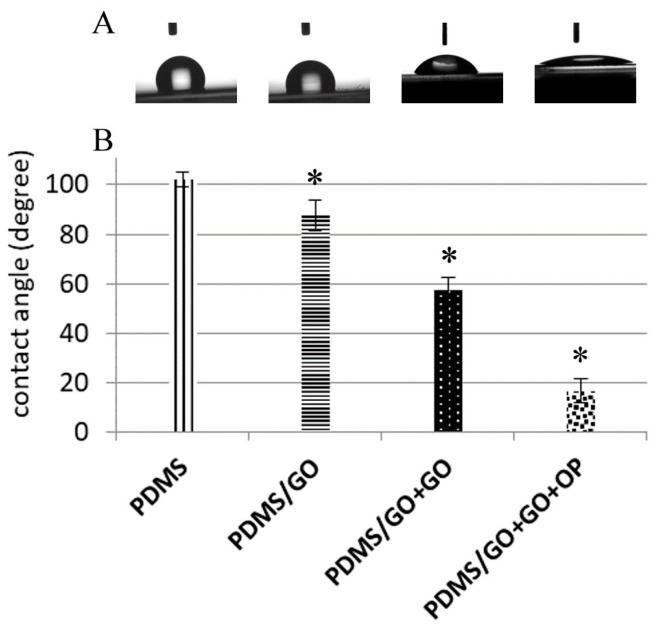
Measurement of water contact angle among pure PDMS and other GO-modified PDMS elastomers. (**A**) Representative images of water drops on the surface of PDMS, PDMS/GO, PDMS/GO + GO, and PDMS/GO + GO exposed to oxygen plasma treatment (PDMS/GO + GO + OP). (**B**) Water contact angle among pure PDMS, PDMS/GO, PDMS/GO + GO, and PDMS/GO + GO with exposure to oxygen plasma treatment were measured by a sessile drop tensiometer. * *p* < 0.05 vs. PDMS. The results are the mean ± SD of three independent experiments. * *p* < 0.05 vs. PDMS.

Previous studies have shown that GO enrichment provides higher physical strength of bioscaffolds [38] and that GO coating onto the substrate also serves as an ideal interface for cell-to-scaffold interaction [31]. After the manufacture of various GO-modified PDMS elastomers, we further measured the elastic modulus and the water contact angle of pure PDMS and the various GO-modified PDMS elastomers (PDMG/GO and PDMS/GO + GO). Compared with pure PDMS, both PDMS/GO and PDMS/GO + GO exhibited significantly higher elastic moduli (Figure 2). At the same time, pure PDMS was characterized by the highest water contact angle, whereas GO enrichment (PDMS/GO) mildly decreased the water contact angle, and the GO coating (PDMS/GO + GO) further suppressed this parameter (Figure 3A). Exposure of the PDMS/GO + GO to oxygen plasma (PDMS/GO + GO + OP) caused the maximal inhibition of water contact angle (Figure 3B). Taken together, these results indicated that the incorporation of GO into PDMS significantly increased the elastic modulus, while the GO enrichment and coating substantially decreased the hydrophobicity of the pure PDMS scaffold. 

### 2.3. Introduction of Hexagonal Micropillars onto the GO-Enriched and GO-Coated PDMS Scaffolds 

**Figure 4:**
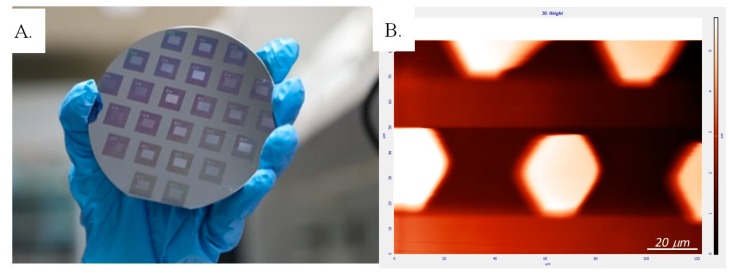
Introduction of hexagonal micropillars onto the PDMS/GO + GO scaffold. (**A**) The wafer molds used to generate the hexagonal micropillar pattern on the scaffolds. (**B**) Atomic force microscopy (AFM) image of PDMS/GO + GO membrane demonstrating the regularly arranged hexagonal micropillars (light areas) lining up on the back side of the PDMS membrane with approximately 5 µm height.

Scaffold fixation in damaged tissue is one of the prerequisites of successful transplantation. Stable positioning of transplants enhances the cell-to-microenvironment interaction, cell biological activity, and cell survival [40]. In the present study, we sought to incorporate suture-free fixation capacity into the developed GO-enriched PDMS scaffold with GO coating (PDMS/GO + GO) for application in nonsuturable tissue. We modified the material into a micropatterned 3D scaffold containing hexagonal pillars by photolithographing on wafer molds (Figure 4A). The wafer molds that were utilized contained regularly arranged small rectangles of approximately 4 mm in width and 6 mm in length. Therefore, in one spin-coating process, a wafer could introduce several patches of hexagonal micropillars onto the PDMS/GO + GO membranes. The surface topography of each scaffold after the micropillar generation was visualized by atomic force microscopy (AFM) (Figure 4B). The representative alternating current amplitude mode AFM images showed the light hexagons and the dark areas surrounding them. The light areas were represented by elevated parts (i.e., the micropillars) on the PDMS/GO + GO membrane, while the darker areas represented the flat valleys between the pillars. The hexagonal micropillars were regularly arranged and were of approximately 5 µm height (Figure 4B). Different sizes and topographic resolution of micropillars were classified into 4 groups, 4_2, 4_8, 8_8, and 10_4, in which the first number represented the 1:10 µm proportion diameter (length between opposite corner) and the second the interval lengths between each pillar.

**Figure 5:**
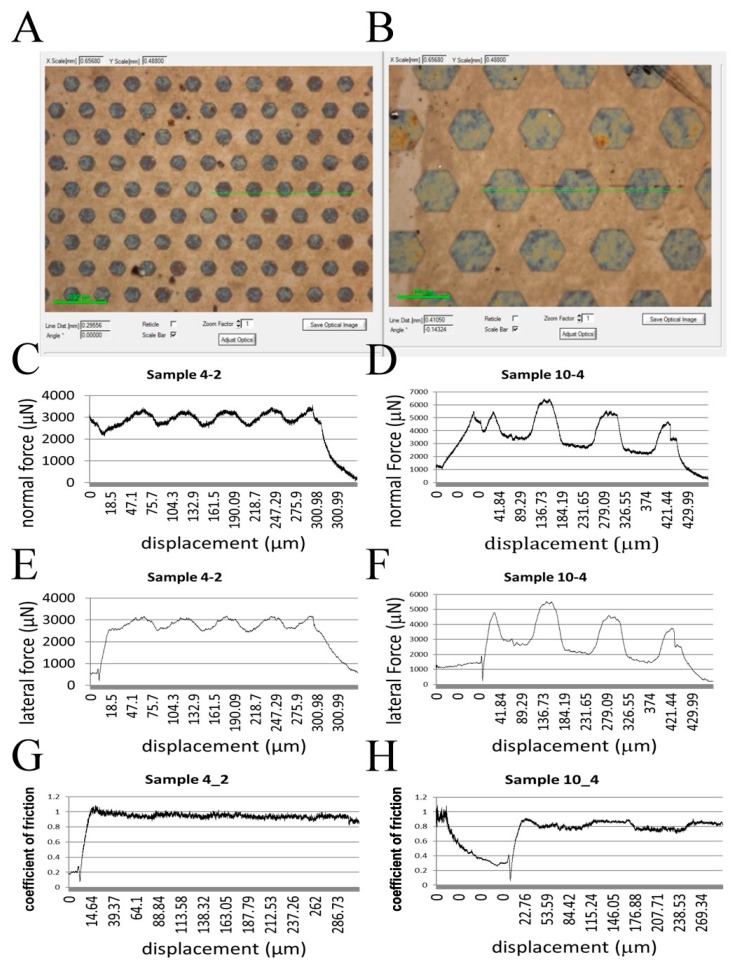
Comparison of normal forces, lateral forces, and coefficient of friction between micropillars of sample 4_2 and sample 10_4. The arrangement and size of the micropillars from sample 4_2 (**A**) and sample 10_4 (**B**). The normal forces (**C**,**D**), lateral forces (**E**,**F**), and coefficient of friction (**G**,**H**) were obtained during the scratch test. As shown in panel G, sample 4_2 shows a higher coefficient of friction than sample 10_4.

After the introduction of hexagonal micropillars onto the PDMS/GO + GO scaffold, we next evaluated the motion resistance of the scaffold by conducting the scratch test. PDMS/GO + GO scaffold with hexogonal micropillars of 10_4 and 4_2 (Figure 5A,B) were tested, and the corresponding normal force and lateral force were measured (Figure 5C–F). The coefficient of friction was calculated by dividing the lateral force by normal force. The results indicated that the detected coefficient of friction during the scratching onto the sample 4_2 surface was significantly higher than that of the scratching onto the sample 10_4 surface. These data revealed that the hexagonal micropillars of the 4_2 surface exhibited higher motion resistance, and therefore may ensure better scaffold fixation than the pillars with other diameters and intervals.

### 2.4. Physical Characteristics of the GO-Enriched and GO-Coated PDMS Scaffold with Hexagonal Micropillars

**Figure 6:**
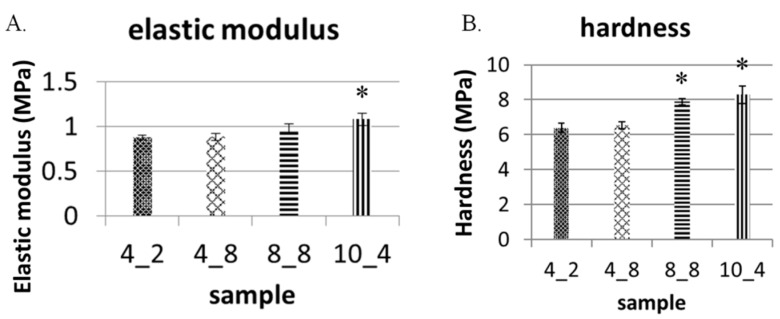
Mechanical strength measurement of PDMS/GO + GO scaffolds with different hexagonal micropillars. Elastic modulus and hardness were measured by nanoindentation. The bar charts showing elastic moduli (**A**) and hardness (**B**) of four classes of GO-coated PDMS scaffolds with hexagonal micropillars. The results are the mean ± SD of three independent experiments. In panels A and B, * *p* < 0.05 versus sample 4_2.

After showing that PDMS/GO + GO scaffolds with smaller hexagonal micropillars were characterized by higher motion resistance, we next analyzed several physical characteristics of PDMS/GO + GO scaffolds with different classes of hexagonal micropillars. Elastic moduli and hardness were measured by the nanoindentation, and it was shown that micropillars of larger size and interval conferred mildly higher elastic moduli and hardness, i.e., mildly elevating the mechanical strength of the surface (Figure 6A,B).

**Figure 7:**
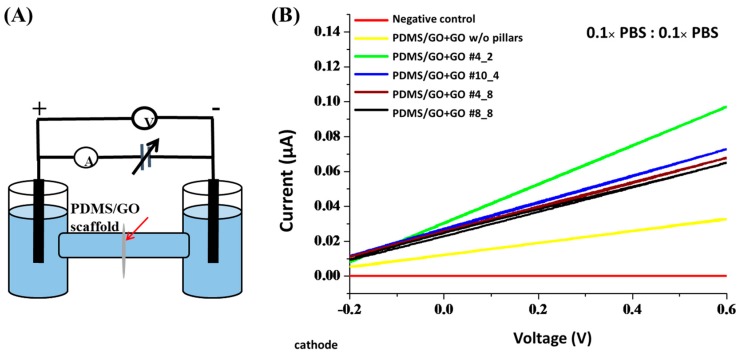
Permeability measurements of the PDMS/GO + GO scaffolds with generation of different micropillars. (**A**) Illustration of the equipment setting for the permeability measurement of PDMS/GO scaffolds. The membrane samples were placed between the cathode (−) and anode (+) chamber. w/o: without. (**B**) The current (I)-voltage (V) curves of PDMS/GO + GO membranes.

The scaffolds used in tissue engineering should be highly permeable in order to support mass transfer [41]. The ion permeability and mass transfer play crucial roles in nutrient diffusion and metabolic waste draining and are therefore important prerequisites of cell health and microenvironment maintenance. Therefore, we sought to evaluate the ion permeability of the PDMS/GO + GO scaffolds with or without hexagonal micropillars by diffusion potential and electric resistivity measurement (Figure 7). The plot of the current response to the voltage increase is shown in Figure 7B. Plastic film used as a negative control showed the highest resistance, with no elevation upon increasing voltage (zero slope) (Figure 7B). The PDMS/GO + GO without pillars demonstrated an elevated slope of current, which reached 0.03 μA, corresponding to 0.6 V of applied voltage. The PDMS/GO + GO membrane with micropillars showed a slightly higher slope. The scaffold with 4_2 micropillars produced the steepest slope of current, which reached approximately 0.1 μA in response to the voltage of 0.6 V (Figure 7B). Among all tested membranes with micropillars, the PDMS/GO + GO scaffold with the finest micropillars (4_2) exhibited the highest permeability, and the negative control (i.e., only plastic film) had the highest resistance (Figure 7B). Collectively, our data indicated that the generation of hexagonal micropillars positively affected the ion permeability of the scaffold.

### 2.5. Biocompatibility of PDMS/GO Scaffold with hiPSC-RPE Cells 

**Figure 8:**
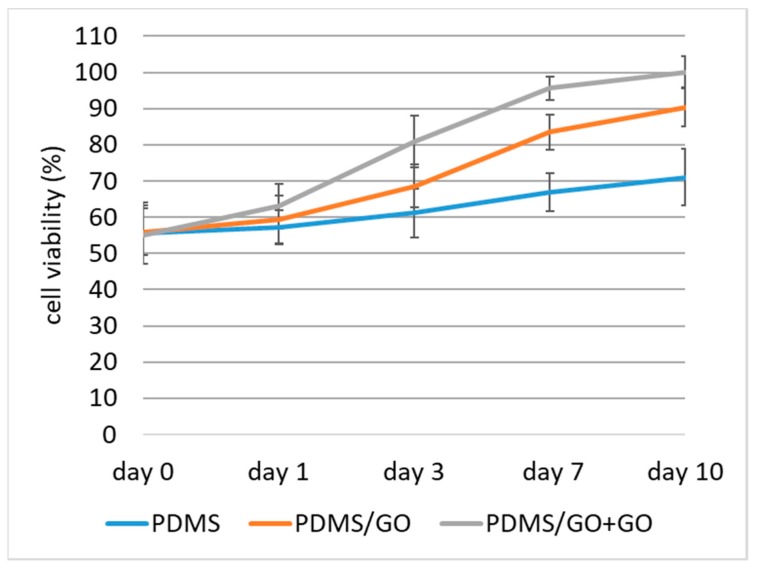
Viability of human induced pluripotent stem cell-derived retinal pigment epithelium cells (hiPSC-RPEs) cultivated on pure PDMS with or without GO enrichment and coating. The cell proliferation was evaluated by using the MTT colorimetric assay, and the cell viability of hiPSC-RPEs cultivated on different scaffolds were measured during a 7-day time course. A stimulatory effect of both GO enrichment and coating on cell survival was observed. PDMS/GO + GO exhibited the optimal cell viability compared to other groups.

Retinal pigment epithelium (RPE) transplantation has become a promising therapeutic method for several retinal diseases, such as age-related macular degeneration (AMD), and several scaffold models have been developed for this purpose [41,42,43,44,45,46]. To validate the biocompatibility of our developed PDMS/GO + GO scaffolds with defined micropillars for cell culture, we cultivated human induced pluripotent stem cell-derived retinal pigment epithelial cells (hiPSC-RPEs). We seeded these cells onto pure PDMS scaffolds with or without modifications. The cell proliferation was evaluated by using the MTT colorimetric assay, and the cell viability of hiPSC-RPEs cultivated on different scaffolds was measured during a seven-day experimental time course (Figure 8). Compared with pure PDMS elastomer only, PDMS with GO enrichment significantly improved the cell viability of hiPSC-RPEs, and the additional GO coating on the scaffold further increased the viability (an upward shift of the cell growth curve was observed; Figure 8). These data indicated that the PDMS membrane with GO enrichment and coating was the ideal scaffold for the cultivation of hiPSC-RPEs.

**Figure 9:**
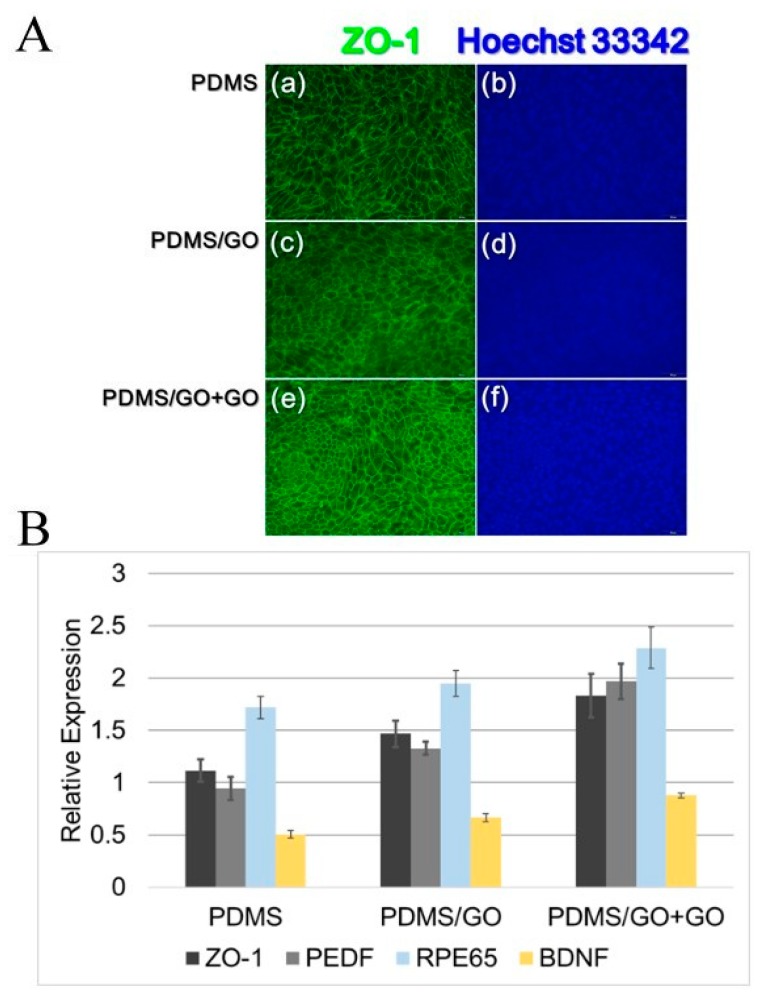
Examination of the expression of ZO-1 protein and various RPE-related genes in hiPSC-RPEs cultivated on PDMS, PDMS/GO, and PDMS/GO + GO. (**A**) Expression of tight junction protein ZO-1 in hiPSC-RPEs cultivated on PDMS membrane with different GO modifications. Immunofluorescence staining was performed to evaluate the ZO-1 expression in hiPSC-RPEs cultivated onto (**a**) pure PDMS, (**c**) PDMS/GO, and (**e**) PDMS/GO + GO. Corresponding Hoechst counterstaining for each treatment is shown (**b**,**d**,**f**). Regular hiPSC-RPE morphology was observed among all conditions, while the PDMS/GO + GO group showed the highest ZO-1 expression. (**B**) Quantitative RT-PCR results revealing the higher expression of RPE-related genes, including ZO-1, PEDF, RPE65, and BDNF, in hiPSC-RPEs cultivated with PDMS with GO modifications and coating.

To further examine the biocompatibility of established biomembranes, we performed immunofluorescence staining to evaluate the expression of tight junction protein zonula occludens-1 (ZO-1) on the hiPSC-RPEs cultivated onto PDMS with various modifications (Figure 9). Compared to the hiPSC-RPE grown on pure PDMS only, there was no obvious effect of the GO enrichment on the ZO-1 expression (PDMS/GO, label c), whereas the additional GO coating (PDMS/GO + GO, label e) substantially increased the amount of ZO-1 (Figure 9A). In addition, we also examined several genes that are crucial for RPE functions and physiology using quantitative PCR. Those examined genes included ZO-1, pigment epithelium-derived factor (PEDF), retinal pigmented epithelium-specific protein with molecular mass of 65 kDa (RPE65), and brain-derived neurotrophic factor (BDNF). Compared with the expression in hiPSC-RPEs cultivated on pure PDMS, hiPSC-RPEs cultivated on PDMS/GO showed higher expression of ZO-1, PEDF, RPE65, and BDNF (Figure 9B). Among these three membranes, hiPSC-RPEs cultivated on PDMS/GO + GO appeared to exhibit the highest expression of these RPE-related genes (Figure 9B). Along with the data of cell viability (Figure 8), our findings particularly indicated that pure PDMS membrane with GO enrichment and coating conferred high biocompatibility and provided an ideal interface for the hiPSC-RPE attachment, viability, and the maintenance of regular morphology. 

## 3. Discussion

Interest in the design, fabrication, and modification of biomimetic scaffolds for tissue engineering has been growing. PDMS, a silicone-based polymer, is one of the most versatile and desirable biomaterials used in surgical prostheses, contact lenses, and microfluidic systems. However, the intrinsic surface hydrophobicity of PDMS limits its application as a scaffold for cell culture as it has negative impact on cell adhesion and expansion, resulting in several abnormalities of cell morphology and attachment [5,13,14]. In addition, the low mechanical strength and unstable fixation of the PDMS scaffold may further hinder its applicability for transplantation. These limitations necessitate the development of novel PDMS membranes with various modifications for minimizing these disadvantages in cell culture and transplantation. 

Stiffness and other mechanical properties of PDMS scaffolds are dependent on the proportion of the mixture of the polymer base and curing agent [47], as well as the curing temperature [48]. PDMS at 10:1 (polymer base to curing agent) proportion has an elastic modulus range of 2.6–2.7 MPa [47]. The data obtained from the variation of curing temperature indicated the Young’s modulus of 1.32–2.97 MPa and hardness of 44–54 Sh_A_ (approximately 0.39–0.63 MPa) [48]. In the past few years, GO has been recognized as a rising star in nanomaterial science due to its aforementioned excellent versatile properties [20,21], including tunable mechanical strength and easy fabrication and functionalization. Previously, it has been shown that the incorporation of a low content of graphene oxide nanoribbon into hydroxyl-terminated PDMS significantly improves the tensile and tear strengths of PDMS [17]. Consistent with these previous findings, our data also revealed that the elastic modulus of the PDMS/GO scaffold was increased compared to the pure PDMS scaffold, and the additional GO coating further increased it (Figure 1). The intrinsic hydrophobic surface of the PDMS elastomer and subsequent low physical adsorption of extracellular matrix component impedes cell attachment and surface functionalization, and leads to cell agglomeration and detachment [5,13,49,50]. Several experimental approaches have been utilized to decrease the hydrophobicity of the biomaterial surface to enhance cell adhesion to the surface [51,52]. As detected by the measurements of water contact angle, our data showed that both GO enrichment and coating led to the reduction of the hydrophobicity of the PDMS elastomer (Figure 2). In addition to these modifications, the hydrophobicity was further suppressed by oxygen plasma treatment. Collectively, GO enrichment and coating increased the elastic modulus and largely improved the hydrophilicity of the PDMS elastomer.

One of the challenges in transplanting a synthetic scaffold carrying a cell sheet is to stabilize it in the transplanted site. A number of studies have attempted to improve the stable fixation of scaffolds using different methods including fibrin glue or suturing [53,54,55,56]. However, limitations exist in nonsuturable areas, particularly in constantly moving organs, such as blinking eyes. Retina transplantation requires a not only biocompatible, but also stable vehicle to deliver cell sheets into the damaged layer. The majority of bioscaffolds developed for retina transplantation are ultrasoft in order to prevent any irritation. Unexpectedly, these characteristics of bioscaffolds may induce folding, flipping, and instability under the incessant movement of the eye. In this study, we designed a slip-resistant surface of a bioscaffold for retina transplantation by inventing a hexagonal micropillar structure at the bottom surface of the PDMS/GO scaffold. We demonstrated that different sizes of pillars and different intervals between them impact the mechanical strength. The finest pillars had higher motion resistance force when compared to the larger pillars. In addition, the connection between the scaffold’s permeability and ion mass transfer, which affects the nutrient supply of the cell, has been reported [41]. In our previous study, moderate membrane resistance and low ion permeability were detected in pure PDMS (current <0.03 µA in response to the voltage of 0.6 V). In the present study, introduction of the micropillar structure on PDMS/GO + GO surged the current response up to 0.1 µA (voltage 0.6 V), suggesting the beneficial effect of the micropillar structure in increasing ion permeability of the PDMS scaffold with GO modifications. 

**Figure 10:**
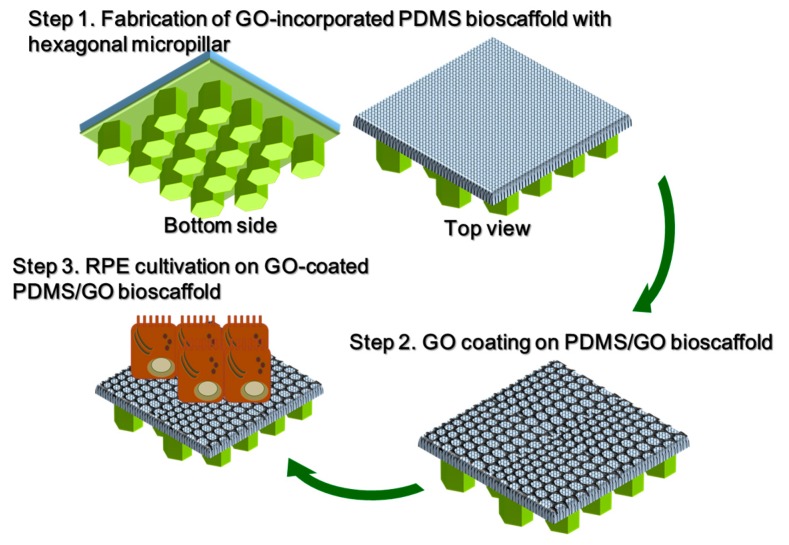
Illustrative scheme for the development of the PDMS/GO + GO bioscaffold with micropillars. First, the PDMS/GO membrane is produced by spin-coating PDMS with GO enrichment on a wafer mold to construct the hexagonal micropillar structure on the bottom surface of the PDMS/GO membrane. Second, the hexagonal micropillar-containing PDMS/GO membranes will be coated with GO to enhance surface hydrophilicity and cell affinity. Afterwards, hiPSC-RPEs derived from human iPSCs are seeded on the PDMS/GO + GO scaffold.

Cultivation of hiPSC-RPEs on the GO-coated PDMS/GO scaffold revealed that such a modified PDMS elastomer provided an ideal interface for normal cell attachment and the maintenance of regular cell growth and viability. ZO-1 has been known as an important tight junction protein of healthy RPE, and detecting high ZO-1 expression in attached hiPSC-RPEs implies that the GO-coated PDMS/GO membrane has high biocompatibility (Figure 10). Further long-term cultivation of hiPSC-RPEs and transplantation studies are required for testing the applicability of such PDMS scaffolds with GO modifications and introduction of micropillars.

## 4. Materials and Methods 

### 4.1. Chemicals and Materials

Polydimethylsiloxane (PDMS; Sylgard^®^ 184), containing polymer base and curing agent, was purchased from Dow Corning, Auburn, MI, USA. Graphene oxide solution (2 wt %) was purchased from US Research Nanomaterials, Inc., (Houston, TX, USA). Natural mouse laminin was purchased from Thermo Fisher Scientific (Melbourne, Australia). All cell culture media were purchased from Thermo Fisher Scientific (Waltham, MA USA). 

### 4.2. Fabrication of PDMS/GO Membrane 

Polydimethylsiloxane (PDMS) membranes were generated using a widely-employed silicone elastomer kit (Sylgard^®^ 184, Dow Corning, Auburn, MI, USA). The PDMS used in the present work is a liquid bicomponent silicone prepolymer. The liquid silicone mixture containing base and curing agent was first mixed. After that, graphene oxide solution was mixed at the amount of 20 wt % of precured PDMS polymer and agitated with a cell spatula to thoroughly mix the graphene oxide with PDMS, which turned a pale-brown colour. The PDMS-GO precured polymer was spin-coated on the wafer mold, which had been immersed in octyltriethoxysilane (Sigma, St. Louis, MO, USA) for the capability of removing PDMS. The PDMS-GO precured polymer was degassed under vacuum to remove air bubbles and baked in an oven at 70 °C for 3 h to thermally cure the PDMS/GO polymeric membrane. 

### 4.3. PDMS Scaffold Surface Modification

For PDMS surface modification, PDMS/GO surface was first hydrophilized by oxygen plasma treatment (PC150, JunSun Tech Co., Ltd., New Taipei City, Taiwan). After surface hydrophilicity increased, the graphene oxide solution was spin-coated on the PDMS/GO membrane with the original concentration (2 wt %) and dried at room temperature. The membrane was cleaned with deionized water (DI water) to remove coated residues and rinsed in PBS before the cell seeding.

### 4.4. Atomic Force Microscopy

An atomic force microscope (NT-MDT spectrum instrument, Tempe, AZ, USA) was utilized to characterize the topography of the generated micropillar scaffold. Surface topography AFM imagery was captured in contact mode with the detection of root mean square (RMS) amplitude of the cantilever as a feedback signal operating under ambient atmosphere on the membrane. A single crystal silicon probe was used with a nominal force constant of around 0.03 N/m and cantilever length of 350 μm. The cantilever resonance frequency was about 9.8 kHz. Images were acquired at a size of 120 μm × 80 μm.

### 4.5. Contact Angle Measurement

Water contact angles on PDMS surfaces were measured at ambient temperature by a sessile drop tensiometer (Model 100SB, Sindatek Instruments Co., Ltd., Taipei, Taiwan). Two microlitres of DI water was dropped on the substrate surface and photographed under optimal backscattered light. The contour of the drop and baseline was then fitted by conic section analysis to calculate the three-phase (solid–liquid–gas) contact point. For each PDMS or PDMS/GO substrate, the measurements were performed near the edge of a sample to acquire clear charge-coupled device (CCD) camera focusing. The water contact angle measurements were presented as means and standard deviations (*n* = 5).

### 4.6. FTIR Characterization

The chemical composition of the generated PDMS/GO scaffolds was investigated using Fourier transform infrared spectroscopy (FTIR; Brookhaven, NY, USA) with a scan size (resolution) of 4 cm^−1^ collected over 256 scans per sample. 

### 4.7. Mechanical Properties Measurement with Nanoindentation

Nanoindentation measurement was performed on a Hysitron Ti 950 Triboindenter (Hysitron, Inc., Minneapolis, MN, USA) loaded with a standard diamond Berkovich indentation tip (highest indentation load: 10 mN). Hardness was determined from an average of 4 indentation measurements for each sample. The elastic modulus was obtained from the measured load-displacement curve during the unloading process and calculated with the Oliver–Pharr method. To verify the mechanical characteristics, as well as adhesive properties of the different micropillar designs, we used scratch methodology. The scratch procedure included the following steps: First, surface profilometry; second, indentation or loading; third, scratching; and last, unloading. The scratch length was 300 μm, with 7.5 μm s^−1^ linear velocity. The scratch tests revealed normal force, lateral force, and coefficient of friction relative to lateral displacement.

### 4.8. Membrane Resistance Analysis (Ion Permeability Test)

The ion permeability test was carried out with a lab-built instrument. The PDMS/GO and membrane sample were clamped between two chambers filled with PBS solution. The ion permeability was measured as the current change relative to the applied voltage. 

### 4.9. Human Induced Pluripotent Stem Cells (hiPSCs) Cultivation and Differentiation to RPEs

This research followed the tenets of the Declaration of Helsinki, and the protocols and procedures were approved by the board of the Taipei Veterans General Hospital. All samples were obtained after patients had given informed consent. iPSCs were created from the T cells isolated from patients’ peripheral blood (10 mL). The hiPSCs were differentiated to hRPEs following Dr. Dennis O. Clegg’s previously established protocol [57]. The hiPSC clumps were first incubated in mTeSR1 culture medium (STEMCELL Technology). When the hiPSCs reached 80% confluence after 5 days, the culture medium was replaced with DMEM/F12 supplemented with 1 × B-27 supplement (Thermo Fisher Scientific), 1 × N2 supplement (Thermo Fisher Scientific), and 1 × nonessential amino acids (NEAA; Invitrogen, Carlsbad, CA, USA). From days 0 to 2, 50 ng/mL Noggin, 10 ng/mL recombinant Dickkopf-related protein 1 (DKK1; Sigma Aldrich, St. Louis, MO, USA), and 10 ng/mL IGF1 (R&D Systems Inc., Minneapolis, MN, USA) were added to the base medium. From days 2 to 4, 10 ng/mL Noggin, 10 ng/mL DKK1, 10 ng/mL IGF1, and 5 ng/mL bFGF were added to the base medium. From days 4 to 6, 10 ng/mL DKK1, 10 ng/mL IGF1 and 100 ng/mL Activin A (R&D Systems Inc., Minneapolis, MN, USA) were added to the base medium. From days 6 to 14, 100 ng/mL Activin A and 10 M SU5402 (EMD Millipore, Burlington, MA, USA) were added to the base medium. After day 14, Activin A was added to the base medium every two days until day 30 and cells were cultured until the black areas indicating RPE formation stopped growing at around day 30. The cells were mechanically enriched by manual selection and collection of the pigmented areas under a dissection microscope. Subsequently, the selected patches of cells were digested using TrypLE Express (Invitrogen, Carlsbad, CA, USA) in a microcentrifuge tube. The TrypLE Express was washed with RPE medium twice and filtered through a cell strainer filter (40 µm, Greiner Bio One, Kremsmünster, Austria) to remove undigested cell clumps, and cells were seeded on Geltrex (Thermo Fisher Scientific)-coated culture dishes. The enriched RPE cells were maintained in RPE medium (DMEM-high glucose supplemented with 3% FBS, 1 × B-27, 1 × penicillin/streptomycin, 1 × l-glutamate and 1 × sodium pyruvate).

### 4.10. Immunofluorescence Staining

hiPSC-RPE cells were seeded onto different surfaces and incubated for 24 h. Cells were then washed twice in PBS, fixed in 4% paraformaldehyde, permeabilized in 0.1% Triton X-100, and blocked in 5% fetal bovine serum–PBS. Cells were incubated with 5 mg/mL of rabbit polyclonal anti-ZO-1 antibody (Thermo Fisher Scientific) and incubated for 1 h. After three washes in PBS, the cells were incubated with goat anti-rabbit secondary antibody (Alexa fluor^®^ 488 conjugated). DAPI was used as nuclear stain (blue). Images were obtained using fluorescent microscopy and a digital camera.

### 4.11. Quantitative RT-PCR

The hRPE cells were collected and the total RNA was isolated by TRIzol reagent (Ambion, Austin, TX, USA) according to the manufacturer’s protocols. One microgram of RNA was prepared for reverse transcription using Superscript III (Invitrogen) to synthesize complementary DNA strands (cDNA). Quantitative PCR was performed using power SYBR green PCR master mix (ABI, Waltham, MA USA) according to the product’s instructions. The real-time PCR reaction was performed and monitored on a step one fast real-time PCR System (Applied Biosystems, Foster City, CA, USA). The primer sequences are listed below. The glyceraldehyde-3-phosphate dehydrogenase (GAPDH) was used as the housekeeping gene, and relative gene expression of target genes was determined using the 2^−ΔΔ *C*t^ method. The primers (both forward and reverse sequences) for ZO-1, PEDF, RPE65, and BDNF are listed as below. ZO-1: CAACATACAGTGACGCTTCACA (forward sequence), GACGTTTCCCCACTCTGAAAA (reverse sequence). PEDF: TTACGAAGGCGAAGTCACCA (forward sequence), TAAGGTGATAGTCCAGCGGG (reverse sequence). RPE65: ACCCAGTGGGGGAAGATTAC (forward sequence), GCAGCAGAGATCCACAATCA (reverse sequence). BDNF: CTACGAGACCAAGTGCAATCC (forward sequence), AATCGCCAGCCAATTCTCTTT (reverse sequence).

### 4.12. Statistical Analysis

The results were demonstrated as the mean ± standard deviation. The differences between groups were analyzed on GraphPad Prism5 version 5.01, using one-way ANOVA followed by Student’s *t*-test. *p*-values < 0.05 were considered statistically significant. 

## 5. Conclusions

In conclusion, we successfully established a novel modified PDMS-based scaffold with GO enrichment, GO coating, and a hexagonal micropillar pattern to improve the mechanical strength and enhance the anchorage stability. We demonstrated that GO incorporation increased the mechanical strength without interrupting the biocompatibility of the PDMS scaffold. Furthermore, a novel hexagonal micropillar pattern on the bottom of the scaffold that had higher motion resistance and ion permeability was established, and it may provide stable fixation of the scaffold without additional fixation required, such as via fibrin glue and suturing. Taken together, this novel scaffold may be suitable for long-term transplantation without sutures and promote the clinical engraftment application as well as fixed position of transplants.

## Abbreviations

PDMS	polydimethylsiloxane
GO	graphene oxide
hiPSC	human induced pluripotent stem cell
hiPSC-RPEs	hiPSC-differentiated retinal pigment epithelial cells
ZO-1	zonula occludens-1
PEDF	pigment epithelium-derived factor
RPE65	retinal pigmented epithelium-specific protein with molecular mass 65 kDa
BDNF	brain-derived neurotrophic factor
CCD	charge-coupled device
NEAA	nonessential amino acids
DKK1	recombinant Dickkopf-related protein 1
